# Allelopathy and Identification of Five Allelochemicals in the Leaves of the Aromatic Medicinal Tree *Aegle marmelos* (L.) Correa

**DOI:** 10.3390/plants13040559

**Published:** 2024-02-19

**Authors:** Seinn Moh Moh, Shunya Tojo, Toshiaki Teruya, Hisashi Kato-Noguchi

**Affiliations:** 1 Department of Applied Biological Science, Faculty of Agriculture, Kagawa University, Miki 761-0795, Kagawa, Japan; hanmohmohyau@gmail.com; 2 The United Graduate School of Agricultural Sciences, Ehime University, Matsuyama 790-8566, Ehime, Japan; 3 Graduate School of Engineering and Science, University of the Ryukyus, 1 Senbaru, Nishihara 903-0213, Okinawa, Japan; k238603@eve.u-ryukyu.ac.jp; 4 Faculty of Education, University of the Ryukyus, 1 Senbaru, Nishihara 903-0213, Okinawa, Japan; t-teruya@edu.u-ryukyu.ac.jp

**Keywords:** *Aegle marmelos*, aromatic medicinal tree, allelochemicals, (*E*)-4-hydroxycinnamic acid methyl ester, *trans*-cinnamic acid, methyl (*E*)-3′-hydroxyl-4′-methoxycinnamate

## Abstract

*Aegle marmelos* (L.) Correa is an economically and therapeutically valuable tree. It is cultivated as a fruit plant in southeast Asian countries. In this research, we investigated the allelopathy and possible allelochemicals in the leaves of *A. marmelos*. Aqueous methanol extracts of *A. marmelos* exhibited significant inhibitory effects against the growth of *Lepidium sativum*, *Lactuca sativa*, *Medicago sativa*, *Echinochloa crusgalli*, *Lolium multiflorum*, and *Phleum pratense*. Bioassay-directed chromatographic purification of the *A. marmelos* extracts resulted in identifying five active compounds: umbelliferone (**1**), *trans*-ferulic acid (**2**), (*E*)-4-hydroxycinnamic acid methyl ester (**3**), *trans*-cinnamic acid (**4**), and methyl (*E*)-3’-hydroxyl-4’-methoxycinnamate (**5**). The hypocotyl and root growth of *L. sativum* were considerably suppressed by these compounds. Methyl (*E*)-3’-hydroxyl-4’-methoxycinnamate also suppressed the coleoptile and root growth of *E. crusgalli*. The concentrations of these compounds, causing 50% growth reduction (*I*_50_) of *L. sativum*, were in the range of 74.19–785.4 μM. The findings suggest that these isolated compounds might function in the allelopathy of *A. marmelos*.

## 1. Introduction

A major challenge facing the agricultural sector is controlling weed plant species, which negatively affect crop productivity [1]. Herbicides are powerful and effective tools for controlling weeds. However, overuse of agrochemicals for pest control has led to various issues such as environmental pollution, unsafe agricultural products, human health problems [2], and the emergence of resistant weed biotypes [3,4]. Because of these negative concerns, investigating alternative methods of weed control is required [5], and allelopathy appears to be one option [6].

Allelopathy is an ecological phenomenon in which a plant releases allelochemicals into the surrounding environment, causing either direct or indirect, positive or negative effects on other plant species (including microbes) [7]. Allelopathic plants produce these allelochemicals by leaching, root secretion, or microbial degradation [8]. Several secondary metabolites (or allelochemicals), such as phenolic acids, flavonoids, terpenoids, coumarins, alkaloids, and their degraded products, have allelopathic effects and play an important role in the ecosystem [9,10]. The primary allelochemicals in plants are thought to be phenolic acids, which include derivatives of cinnamic and benzoic acids [11,12]. Phenolic compounds are reported in a wide range of plant groups, including trees, crops, weeds, and medicinal plants [13]. Compared with other plants, medicinal plants may contain more bioactive molecules [14]. In addition, strong allelopathic effect has been found in secondary metabolites isolated from medicinal plants [15]. Moreover, phenolic compounds derived from medicinal plants are beneficial to human health [16] and can be applied as allelochemicals for weed management [13]. Research on the allelopathic properties of medicinal plants offers an effective alternative for diversifying weed management approaches. Therefore, the allelopathy of medicinal plants has attracted attention in recent years. Other studies have also confirmed that medicinal plants possess an allelopathic effect and allelopathy-associated substances [13,17,18]. Based on the reported literature, this study assumed that the majority of medicinal plants have allelopathic effects and contain active compounds. 

**Figure 1:**
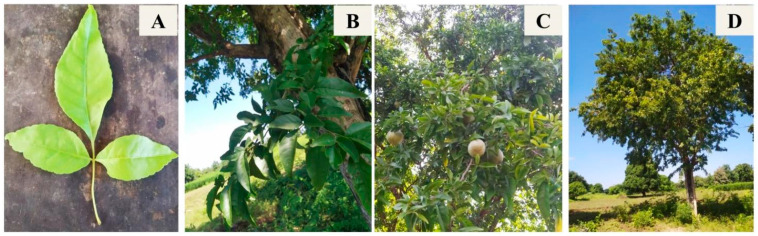
(**A**) Trifoliate leaf, (**B**) stem, (**C**) fruits, and (**D**) tree of *A. marmelos*.

*Aegle marmelos* (L.) Correa, belonging to the Rutaceae family, is highly reputed for its medicinal properties [19]. It is commonly distributed in the dry forests of India, Thailand, Myanmar, Bangladesh, Sri Lanka, and Pakistan [20]. *A. marmelos* is a slow-growing, small- to medium-sized tree around 25 to 30 feet in height [21] (Figure 1). The tree is aromatic, and all its parts are medicinally important [22]. The different plant parts (leaves, fruit, seeds, roots, and bark) are used as folk medicine to treat several ailments and possess anti-inflammatory, antifungal, analgesic, antipyretic, hypoglycemic, antidyslipidemic, immunomodulatory, antiproliferative, anti-fertility, and insecticidal activity [22,23,24]. This tree contains more than one hundred phytochemicals, such as tannins, cardiac glycosides, alkaloids, flavonoids, phenol, terpenoids, and steroids [25]. Although all parts of the tree have been explored for their medicinal properties [26,27] and plant growth inhibitory potential [28,29], there is still a lack of evidence related to their allelochemicals. Thus, the aim of the current study is to evaluate the allelopathic potential of the leaf extracts of *A. marmelos* and to investigate the related allelochemicals.

## 2. Results

### 2.1. Growth-Suppressive Activity of A. marmelos

**Figure 2:**
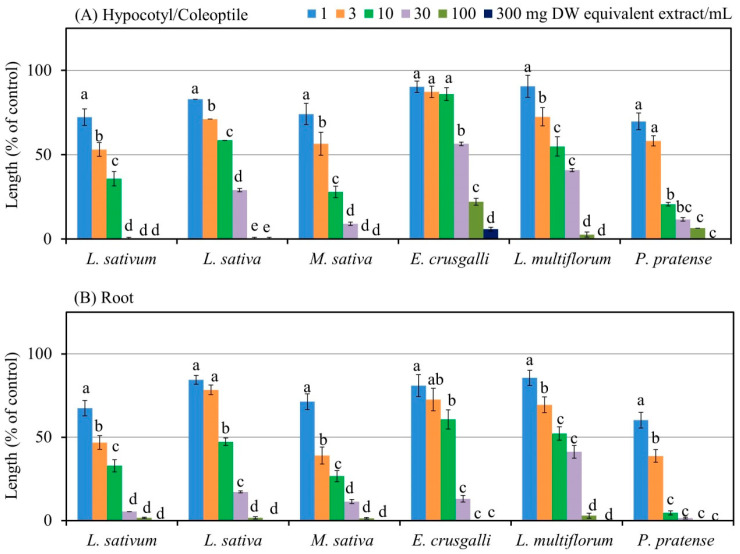
The hypocotyl/coleoptile growth (**A**) and root growth (**B**) of the six examined plants at the different doses of *A. marmelos* leaf extract. The vertical bars express mean ± standard error (SE) (six replicates × 10 seedlings). Different letters specify significant differences between the *A. marmelos* treatments and the control at the 5% probability level (Tukey’s test).

The leaf extracts of *A. marmelos* had significant growth-suppressive effects against the seedling (hypocotyl/coleoptile and root) growth of *L. sativum*, *L. sativa*, *M. sativa*, *E. crusgalli*, *L. multiflorum*, and *P. pratense* at concentrations of more than 10 mg DW equivalent *A. marmelos* extract/mL, except the coleoptile growth of *E. crusgalli* (Figure 2A,B). The concentration of 30 mg DW equivalent *A. marmelos* extract/mL completely inhibited the growth of the *L. sativum* hypocotyls and inhibited the hypocotyl/coleoptile growth of *L. sativa*, *M. sativa*, *E. crusgalli*, *L. multiflorum*, and *P. pratense* to 28.9, 8.9, 56.4, 40.8, and 11.6% of the control, respectively (Figure 2A), whereas the root growth was inhibited to 5.4, 17.2, 11.4, 13.2, 41.2, and 1.5% of the control, respectively (Figure 2B). Moreover, the concentration of 300 mg DW equivalent *A. marmelos* extract/mL completely suppressed the hypocotyl/coleoptile and root lengths of *L. sativum*, *L. sativa*, *M. sativa*, *E. crusgalli*, *L. multiflorum*, and *P. pratense*, except the coleoptile length of *E. crusgalli*, compared with control.

The concentrations of *A. marmelos* extract needed for 50% growth restriction (*I*_50_ values) of the hypocotyl/coleoptile and root growth of the six examined plants (*L. sativum*, *L. sativa*, *M. sativa*, *E. crusgalli*, *L. multiflorum*, and *P. pratense*) were in the ranges of 3.16–36.14 and 1.61–10.06 mg DW equivalent *A. marmelos* extract/mL, respectively (Table 1). The *I*_50_ values for the extract against the hypocotyl/coleoptile growth of the six examined plants were significantly greater than for the root growth. Of the six examined plants, the *I*_50_ values showed that the *P. pratense* coleoptiles and roots were more responsive to the *A. marmelos* extracts, whereas the *E. crusgalli* coleoptiles and *L. multiflorum* roots were least sensitive.

The correlation coefficient values between the dose of *A. marmelos* extract and the seedling growth of the test plants ranged from −0.70 to −0.91 (Table 1).

### 2.2. Identification of the Five Active Compounds

**Figure 3:**
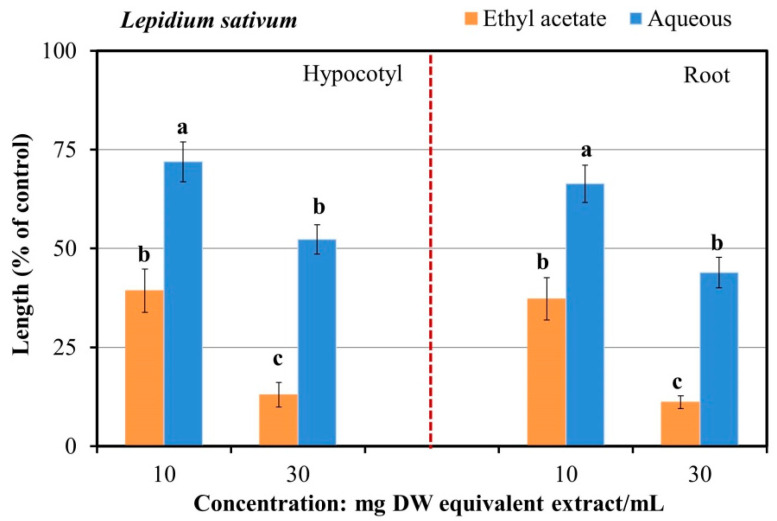
*L. sativum* growth response to the two fractions of *A. marmelos* (ethyl acetate and aqueous) extracts. The different letters specify significant difference at the 5% probability level (Tukey’s test).

The results (Figure 3) showed that, compared with the aqueous fraction, the ethyl acetate fraction notably restricted the growth of the *L. sativum* seedlings (hypocotyl and root) with the application of 10 and 30 mg DW equivalent *A. marmelos* extract/mL. Thus, the ethyl acetate fraction was separated using a sequence of chromatography steps: silica gel, Sephadex LH-20, reverse-phase C_18_ cartridges, and HPLC. Consequently, the five substances with growth-suppressive effects were isolated by reverse-phase HPLC and identified using spectroscopy.

**Figure 4:**
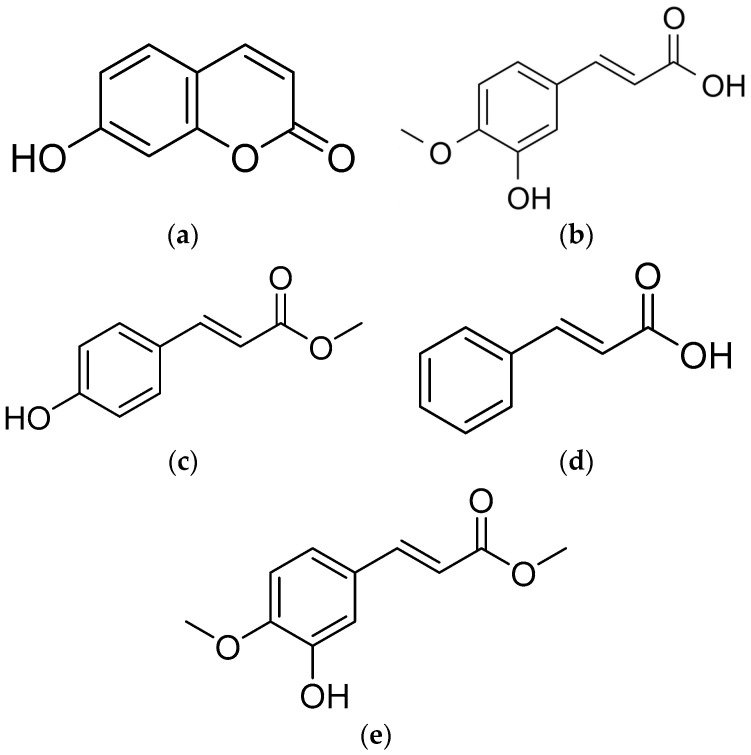
Chemical structures of the five identified compounds. (**a**) Umbelliferone, (**b**) *trans*-ferulic acid, (**c**) (*E*)-4-Hydroxycinnamic acid methyl ester, (**d**) *trans*-cinnamic acid, (**e**) Methyl (*E*)-3′-hydroxyl-4′-methoxycinnamate.

The chemical formula of compound-**1** was determined to be C_9_H_5_O_3_ (isolated as a colorless powder). The ^1^H-NMR spectrum of this compound was determined by 500 MHz, acetone-*d*_6_. The HRESIMS data, ^1^H-NMR spectrum, and optical rotation of this compound are similar to the published literature [30]. By comparing its spectral data with other research [31], this compound was identified as umbelliferone (Figure 4a).

Compound-**2** was isolated as a colorless powder. The molecular formula C_10_H_9_O_4_ was determined by HRESIMS. The ^1^H-NMR spectrum (500 MHz, CD_3_OD + CDCl_3_) showed three aromatic proton signals at *δ*_H_ 7.05 (1H, d, *J* = 1.9), 7.00 (1H, dd, *J* = 8.3, 1.9), and 6.87 (1H, d, *J* = 8.3); one methyl proton signal at δH 3.88 (3H, s); and two olefinic proton signals at *δ*_H_ 7.53 (1H, d, *J* = 15.8) and 6.24 (1H, d, *J* = 15.8). The NMR data of this compound was in agreement with reported data in the literature [32], and the compound was identified as *trans*-ferulic acid (Figure 4b). 

Compound-**3** was isolated as a colorless powder. The molecular formula C_10_H_10_O_3_ was determined by HRESIMS. The ^1^H-NMR spectrum (500 MHz, CD_3_OD) showed four aromatic proton signals at *δ*_H_ 7.46 (2H, d, *J* = 8.6) and 6.81 (2H, d, *J* = 8.6); one methyl proton signal at *δ*_H_ 3.76 (3H, s); and two olefinic proton signals at *δ*_H_ 7.62 (1H, d, *J* = 16.0) and 6.33 (1H, d, *J* = 16.0). The spectral data of compound-2 was in agreement with reported data in the literature [33], and the compound was identified as (*E*)-4-hydroxycinnamic acid methyl ester (Figure 4c).

Compound-**4** was isolated as a colorless powder and the chemical formula C_9_H_8_O_2_ was determined by HRESIMS. The ^1^H-NMR spectrum (500 MHz, CDCl_3_) showed five aromatic proton signals at *δ*_H_ 7.56 (2H, m) and 7.43–7.39 (3H, m) and two olefinic proton signals at *δ*_H_ 7.78 (1H, d, *J* = 16.0) and 6.46 (1H, d, *J* = 16.0). After comparing the spectral data of compound-**3** with that in the published literature [34], this compound was identified as *trans*-cinnamic acid (Figure 4d).

Compound-**5** was isolated as a colorless powder. The molecular formula C_11_H_11_O_4_ was determined by HRESIMS. The ^1^H-NMR spectrum (500 MHz, CDCl_3_) showed three aromatic proton signals at *δ*_H_ 7.13 (1H, d, *J* = 2.1), 7.03 (1H, dd, *J* = 8.3, 2.1), and 6.84 (1H, d, *J* = 8.3); two methyl proton signals at *δ*_H_ 3.93 (3H, s) and 3.79 (3H, s); and two olefinic proton signals at *δ*_H_ 7.60 (1H, d, *J* = 15.9) and 6.29 (1H, d, *J* = 15.9). After comparing these spectral data with information from published documents [35], it was determined that the chemical structure of this compound was methyl (*E*)-3′-hydroxyl -4′-methoxycinnamate (Figure 4e). The spectroscopic data of the five compounds are provided in the Supplementary File.

### 2.3. Allelopathic Effect of the Five Compounds

**Figure 5:**
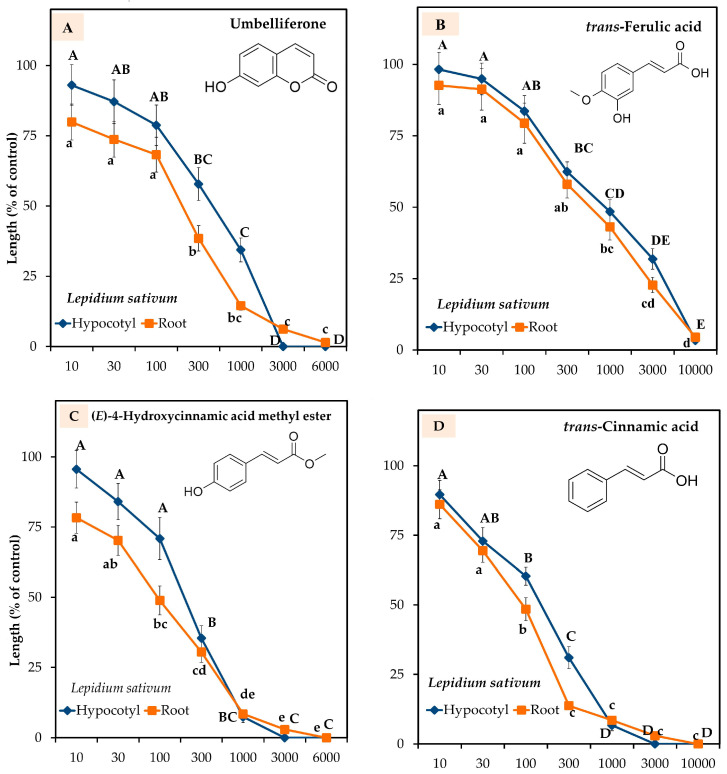

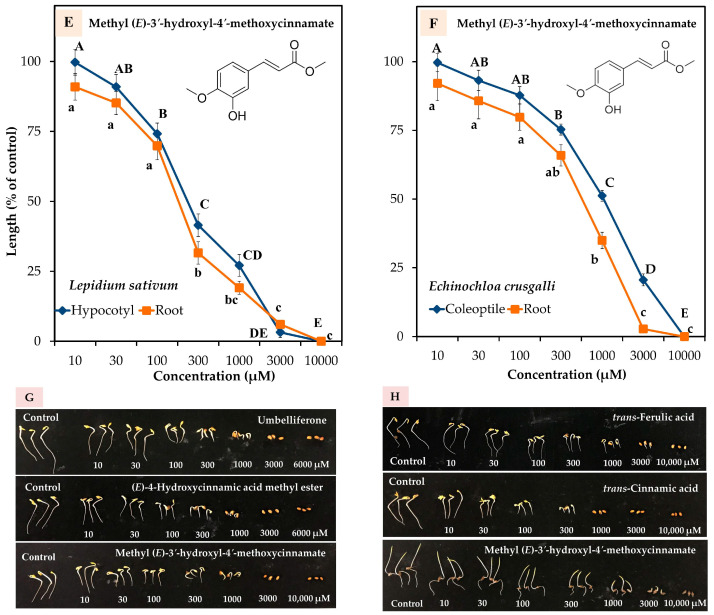
Effects of compound-**1** (**A**), -**2** (**B**), -**3** (**C**), -**4** (**D**), and -**5** (**E**) on the hypocotyl and root growth of *L. sativum*. Effects of compound-**5** (**F**) on the coleoptile and root growth of *E. crusgalli*. Seedling growth of *L. sativum* in response to compound-**1**, -**2**, -**3**, -**4**, and -**5**, and seedling growth of *E. crusgalli* in response to compound-**5** (**G**,**H**) at different of concentrations.

The five characterized compounds significantly suppressed *L. sativum*, and the growth-suppressive effect depended on the concentration of each compound. (*E*)-4-Hydroxycinnamic acid methyl ester (compound-**3**), *trans*-cinnamic acid (compound-**4**), and methyl (*E*)-3′-hydroxyl-4′-methoxycinnamate (compound-**5**) significantly restricted the hypocotyls and roots at extract concentrations of more than 100 µM (Figure 5C–E). At the concentration of 1000 µM, umbelliferone (compound-**1**) significantly inhibited the hypocotyl and root lengths of *L. sativum* to 34.4 and 14.5%, *trans*-ferulic acid (compound-**2**) to 48.5 and 43.1%, compound-**3** to 7.4 and 8.5%, compound-4 to 6.7 and 8.5%, and compound-**5** to 27.1 and 19.2% of the control growth, respectively (Figure 5A,B). Of the five compounds, compound-**5** obtained the highest crude weight, and at the concentration of 10,000 μM, this compound fully suppressed the coleoptile and root lengths of *E. crusgalli* (Figure 5F).

The *I*_50_ values for suppressing the growth of the *L. sativum* hypocotyls and roots were 378.2 and 153.1 µM for compound-1, 785.4 and 552.6 µM for compound-**2**, 179.3 and 83.8 μM for compound-**3**, 119.6 and 74.1 µM for compound-**4**, and 260.5 and 181.8 µM for compound-**5**, respectively (Table 2). The *I*_50_ values of all the compounds for suppressing *L. sativum* hypocotyl growth were 2.4, 1.4, 2.01, 1.6, and 1.4 times larger than for the roots, respectively. The *I*_50_ values of compound-**4** showed more growth allelopathic potential than the remaining four compounds. The *I*_50_ values of the five compounds for suppressing the hypocotyl and root lengths of *L. sativum* ranged from 74.1 to 785.4 µM. The *I*_50_ values of compound **5** for suppressing the coleoptile and root growth of *E. crusgalli* were 885.1 and 453.1 µM, respectively.

## 3. Discussion

The *A. marmelos* extracts exhibited a growth-suppressive effect against the seedling (hypocotyl/coleoptile and root) growth of the dicot (*L. sativum*, *L. sativa*, and *M. sativa*) and monocot plants (*E. crusgalli*, *L. multiflorum*, and *P. pratense*) (Figure 2). Moreover, the correlation coefficients (r) revealed a significant negative relationship between the hypocotyl/coleoptile and root lengths of the bioassayed plant species and the extract concentrations (Table 1). These results suggested that the *A. marmelos* extract concentrations have the growth inhibitory activity of bioassays plants. Additionally, the different *I*_50_ values of the *A. marmelos* extracts for the hypocotyl/coleoptile and root growth of *L. sativum*, *L. sativa*, *M. sativa*, *E. crusgalli*, *L. multiflorum*, and *P. pretense* indicate that the inhibitory effect of the extracts depends on the six examined plant species (Table 1). Similar results of dose- and species-dependent allelopathic properties have been documented by other researchers [36,37,38]. The growth inhibitory activity of the *A. marmelos* extracts against the six plant species investigated in this study indicates that these extracts may involve active compounds that have growth-suppressive properties.

The leaf extracts were separated and loaded via a series of chromatographic steps, and five substances were identified by spectroscopy as umbelliferone, *trans*-ferulic acid, (*E*)-4-hydroxycinnamic acid methyl ester, *trans*-cinnamic acid, and methyl (*E*)-3′-hydroxyl-4′-methoxycinnamate. Umbelliferone, also referred to as 7-hydroxycoumarin, is a member of the coumarin family [39] and is commonly found across several plant families, such as Rutaceae and Umbelliferae [40]. This compound possesses many pharmacological properties such as antioxidant [41], anti-inflammatory [42], and antifungal and anti-bacterial [40] properties. Other studies have described the growth-suppressive activities of umbelliferone against *Vulpia myuros* [30] *L. sativa*, *Eruca sativa*, and *Hordeum vulgare* [43].

*Trans*-ferulic acid (or (*E*)-3-hydroxy-4-methoxycinnamic acid) is a stereoisomer of ferulic acid and is widely present in cereal crops (rice and wheat), fruits (bananas and pineapples), and vegetables (peanuts, eggplants, and tomatoes) [44]. Based on the reports of other studies, this compound has many pharmaceutical properties such as antioxidant [45], and antimicrobial and antifungal [44] properties. Additionally, ferulic acid inhibits the root growth of *Vigna radiata* [46], and the net rate of photosynthesis and stomatal conductance in *Rhododendron delavayi* [47].

*Trans*-cinnamic acid (or (2*E*)-3-phenylprop-2-enoic acid) is a natural aromatic carboxylic acid and is widely present in plants such as *Cinnamomum cassia* and *Panax ginseng*, fruits, whole grains, vegetables, and honey [48]. The antimicrobial, antioxidant, and antifungal properties of *trans*-cinnamic acid have led to its widespread use in food, cosmetics, and pharmaceuticals [49,50]. Moreover, *trans*-cinnamic acid exhibits allelopathic effects on the seed sprouting and seedling growth of *Glycine max* [51], *Lactuca sativa* [52], and *Chrysanthemum coronarium* [53]. *Trans*-cinnamic acid is liberated into the soil through plant tissue decomposition, leaf leachate, and root secretion, e.g., *Elytrigia repens* [51], *Cucumis sativus* [52], and *Medicago sativa* [53]. Anh et al. (2021) [54] reported that *trans*-cinnamic acid inhibits *Glycine max* seedlings through the disorder and interference of the uptake and transport of ions in the plant and, consequently, causes morphological distortion of the roots.

(*E*)-4-Hydroxycinnamic acid methyl ester and methyl (*E*)-3′-hydroxyl -4′-methoxycinnamate are part of the group of hydroxycinnamic acids (HCAs) that are derivatives of phenolic compounds [55,56]. *Trans*-cinnamic acid derivatives that include HCAs are reported to have anti-bacterial, anti-inflammatory, and antifungal activities [55,57]. Whole grains, coffee, tea leaves, fruits, and vegetables are rich sources of HCAs [58,59,60]. DellaGreca et al. (2007) [61] reported that cinnamic ester derivatives obtained from *Oxalis pes-caprae* affect the seedling growth of *L. sativum*. Other studies have found that some phenolic acids affect ion transport and metabolism [62], interfere with cell division and deform cellular structures [63], and reduce photosynthetic rates [64]. Although the phytochemical properties of the characterized compounds from all parts of *A. marmelos* have been described for a long time, this study is the first account of the growth-suppressive activity of umbelliferone, *trans*-ferulic acid, (*E*)-4-hydroxycinnamic acid methyl ester, cinnamic acid, and methyl (*E*)-3′-hydroxyl-4′-methoxycinnamate from *A. marmelos* leaves.

In this study, umbelliferone, *trans*-ferulic acid, (*E*)-4-hydroxycinnamic acid methyl ester, *trans*-cinnamic acid, and methyl (*E*)-3′-hydroxyl-4′-methoxycinnamate significantly reduced the growth of *L. sativum* in a dose-dependent manner (Figure 5A–F). Allelochemicals such as phenols, coumarin, and terpenoids cause lipid peroxidation [65]. In addition, phenolic acids, including *trans*-cinnamic acid, disturb membrane permeability and thus affect plant growth processes [65,66,67]. Comparing *I*_50_ values, *trans*-cinnamic acid exhibited the most allelopathic effects against *L. sativum* (Table 2). The strong growth-suppressive effect of this compound may be linked to its hydrophobicity and the -OH and -OCH_3_ groups in the molecules. Pinho et al. (2017) [68] stated that phenolic compounds appear to be less allelopathic to plants when the number of -OH and -OCH_3_ groups are increased. The *I*_50_ values also showed that the sensitivity to the five compounds was greater against the roots than the hypocotyls. These results indicate that the roots are the first parts to be exposed to the phenolic allelochemicals [69,70].

According to the results of this study, *A. marmelos* leaves possess allelopathic agents (umbelliferone, *trans*-ferulic acid, (*E*)-4-hydroxycinnamic acid methyl ester, *trans*-cinnamic acid, and methyl (*E*)-3′-hydroxyl-4′-methoxycinnamate), which account for the growth-suppressive activity of this tree. Additionally, the fresh and dry aromatic leaves of *A. marmelos* and their extract can be used for living or dead mulch and foliar spray as weed-suppressive resources for controlling weeds. Therefore, the allelopathy of *A. marmelos* and its allelochemicals may be helpful in developing environmentally safer herbicides for weed management, as well as minimizing or eliminating the pollution problems of agrochemicals in the environment. 

## 4. Materials and Methods

### 4.1. Plant Materials and Test Plant Species

*Aegle marmelos* leaves were brought from Khin U Region, Sagaing Division, Myanmar (22°49′4″ N and 95°48′12″ E) during July–August 2020. The leaves were shade dried and ground to obtain fine powder. Before starting the experiment, the powder was retained in plastic bags at 2 °C. To assess the allelopathic potential of *A. marmelos*, three dicot (*L. sativum*, *L. sativa*, and *M. sativa*) and three monocot (*E. crusgalli*, *L. multiflorum*, and *P. pratense*) plant species were selected for bioassays.

### 4.2. Extraction and Growth Bioassay

Dry powdered leaf (250 g) was extracted by soaking in 70% (*v*/*v*) aqueous methanol (1500 mL) for 48 h. The extract was filtered through a single layer of filter paper (No. 2, 125 mm; Toyo Ltd., Tokyo, Japan). After filtration, the extract residue was re-extracted by soaking in methanol (1500 mL) for 24 h and filtered. The two extracts obtained after filtration were then mixed and concentrated in a rotavapor to obtain an aqueous solution at 40 °C. The crude extracts (100 g dry weight) were dissolved in 30 mL of methanol, and various bioassay doses (1 (0.18 μL), 3 (0.54 μL), 10 (1.8 μL), 30 (5.4 μL), 100 (18.2 μL), and 300 (54.5 μL) mg dry weight (DW) equivalent *A. marmelos* extract/mL) were prepared in Petri dishes (2.8 cm). Concentrated extract (600 μL) and control (only methanol) were added to sheets of filter paper (No. 2; Toyo Ltd.) in Petri dishes. The methanol in the Petri dishes was then evaporated in a draft chamber. After drying, 600 μL of a 0.05% aqueous Tween 20 solution (Nacalai Tesque, Inc., Kyoto, Japan) was added to the filter papers. Ten seeds of *L. sativum*, *L. sativa*, and *M. sativa* and ten sprouted seeds (to avoid dormancy of the seed and incubated at 25 °C for 36–48 h) of *E. crusgalli*, *L. multiflorum*, and *P. pratense* were placed in the Petri dishes and incubated at 25 °C for 48 h. The lengths of the hypocotyls and roots of the test plant species were then measured with a ruler and the growth-suppressive potential was determined. The growth assay experiment was carried out using a completely randomized design (CRD) with six replications (10 seedlings/replicate, *n* = 60).

### 4.3. Isolation and Purification of the Active Compounds

As previously described, 3.3 kg of *A. marmelos* leaf powder was extracted and filtered. An *L. sativum* bioassay was used to evaluate the activity of the obtained fractions in each isolation step. The aqueous residue was adjusted using 1 M phosphate buffer to reach pH 7.0 and partitioned six times with ethyl acetate (an equal volume of 150 mL per time). The resulting ethyl acetate fraction (after being concentrated until dryness) was loaded onto a silica gel column (70-230 mesh; Nacalai Tesque), eluted with 20 (F1), 30 (F2), 40 (F3), 50 (F4), 60 (F5), 70 (F6), and 80% (F7) ethyl acetate in n-hexane (*v*/*v*; 150 mL per step), ethyl acetate (150 mL) (F8), and methanol (300 mL) (F9). Of the nine fractions, fraction 5 (F5) showed the highest activity. After the evaporation of F5, the residue was further subjected to a Sephadex LH-20 column (GE Healthcare, Uppsala, Sweden) and eluted with 20 (F1), 40 (F2), 60 (F3), and 80% (F4) aqueous methanol (*v*/*v*; 150 mL per step) and methanol (300 mL) (F5). Of the five fractions, F4 (eluted with 80% aqueous methanol) exhibited the most inhibitory activity. After evaporation of this fraction, the crude residue was loaded to a C_18_ cartridge (1.2 × 6.5 cm; YMC, Kyoto, Japan) and eluted with 20 (F1), 30 (F2), 40 (F3), 50 (F4), 60 (F5), 70 (F6), 80 (F7), and 90% (F8) (*v*/*v*) aqueous methanol (15 mL per step) and methanol (30 mL per step) (F9). Of the nine fractions, F2 and F3 showed the most bioactivity.

Active fraction F2 was purified using reverse-phase HPLC (3 µm, 4.6 × 250 mm i.d., Inertsil ODS-3; GL Science Inc., Tokyo, Japan) with 35% aqueous MeOH (flow rate = 0.8 mL/min). Inhibitory effect was detected in active peak fractions 1 and 2 at the retention times of 22–26 and 38–49 min, respectively, resulting in active compounds **1** and **2**.

Active fraction F3 was also purified using reverse-phase HPLC with 45% aqueous MeOH (flow rate = 0.8 mL/min). Inhibitory effect was detected in active peak fractions 3, 4, and 5 at the retention times of 50–53, 53–56, and 57–60 min, respectively, resulting in active compounds **3**, **4**, and **5**.

The NMR spectral data were documented on a Bruker AVANCE III 500 MHz NMR spectrometer. Chemical shifts were described to the residual signal of solvent (acetone-*d*_6_, CD_3_OD and CDCl_3_). HRESIMS was measured on a Thermo Scientific Orbitrap Exploris 240 Mass Spectrometer.

### 4.4. Bioassays of the Identified Compounds

The identified compounds (**1**, **2**, **3**, **4**, and **5**) were dissolved in 1000 μL of methanol. Assay doses of each compound (10, 30, 100, 300, 1000, 3000, and 6000/10,000 μM) were then prepared and added to filter paper (No. 2) in Petri dishes (2.8 cm). The Petri dishes were placed inside a draft chamber. After the methanol evaporated, the filter papers were moistened with 600 μL Tween 20. Ten uniform seeds of *L. sativum* and 10 sprouted seeds of *E. crusgalli* (to avoid dormancy of the seed and incubated at 25 °C for 48 h) were placed on the filter papers and incubated at 25 °C for 48 h. The bioassays experiment for each test plant included three replicates, each with 10 seedlings (*n* = 30). The hypocotyl/coleoptile and root lengths were then measured by ruler to calculate the percentage of seedling growth as mentioned in Section 2.2 above.

### 4.5. Statistical Analysis

The growth assay experiment was carried out using a CRD with six replications. The results are shown as mean ± SE. ANOVA of all the data was analyzed using SPSS (version 16.0) (SPSS Inc., Chicago, IL, USA). Tukey’s test was used to evaluate the significant differences between the control and treated plant groups at a *p*-value of 0.05. The doses of the *A. marmelos* extracts and each compound needed for 50% growth restriction (*I*_50_) of each examined plant species were calculated using GraphPad Prism 6.0 (Software of GraphPad, Inc., La Jolla, CA, USA).

## 5. Conclusions

The extracts of *A. marmelos* exhibited significant allelopathic potential against the seedlings of the six examined plants (*L. sativum*, *L. sativa*, *M. sativa*, *E. crusgalli*, *L. multiflorum*, and *P. pretense*). Five phenolic compounds with growth-suppressive effects were separated from the *A. marmelos* leaves and characterized as umbelliferone, *trans*-ferulic acid, (*E*)-4-hydroxycinnamic acid methyl ester, *trans*-cinnamic acid, and methyl (*E*)-3′-hydroxyl-4′-methoxycinnamate. These allelochemicals greatly inhibited the hypocotyl and root lengths of *L. sativum*. *Trans*-cinnamic acid showed the most allelopathic effect against *L. sativum*. The growth-suppressive properties of these characterized compounds might be related to *A. marmelos* leaf allelopathy. Therefore, the fresh and dry aromatic leaves of *A. marmelos* might be useful for living and dead mulch as weed-suppressive or soil-additive resources, and its allelochemicals could be useful in developing an allelochemical-based natural herbicide in sustainable agriculture. However, further experiments with the allelopathy of *A. marmelos* and the mode of action of its allelochemicals are necessary to examine in both laboratory and field settings for the development of natural herbicides. 

## Figures and Tables

**Table 1:** The doses of *A. marmelos* extract that suppressed 50% of the hypocotyl/coleoptile and root growth (*I*_50_) and correlation coefficients of the six examined plants by treatment with the *A. marmelos* extracts.

Test Plant Species		*I*_50_ (mg DW Equivalent *A. marmelos* Extract/mL)		Correlation Coefficient ®	
		Hypocotyl/Coleoptile	Root	Hypocotyl/Coleoptile	Root
Dicot	*L. sativum*	3.3	2.6	−0.79 **	−0.79 **
	*L. sativa*	10.0	8.3	−0.83 **	−0.91 **
	*M. sativa*	3.5	2.3	−0.70 **	−0.75 **
Monocot	*E. crusgalli*	36.1	8.9	−0.78 **	−0.71 **
	*L. multiflorum*	12.0	10.6	−0.70 **	−0.77 **
	*P. pratense*	3.1	1.61	−0.76 **	−0.73 **

Asterisks indicate statistical significance at the 0.05% probability level (Tukey’s test).

**Table 2:** The doses of the five identified compounds that suppressed 50% of the hypocotyl and root growth (*I*_50_) of *L. sativum*. The *I*_50_ values of compound-**5** against the coleoptile and root length of *E. crusgalli*.

Identified Compound	*I*_50_ (µM)			
	*L. sativum*		*E. crusgalli*	
	Hypocotyl	Root	Coleoptile	Root
Compound-**1**	378.2	153.1		
Compound-**2**	785.4	552.6		
Compound-**3**	179.3	83.8		
Compound-**4**	119.6	74.1		
Compound-**5**	260.5	181.8	885.1	453.1

## Data Availability

Data will be made available on request.

## Supplementary Materials

The following supporting information can be downloaded at: https://www.mdpi.com/article/10.3390/plants13040559/s1, The Spectral data of five compounds.
