# Supplementary material for: Allelopathy and Identification of Five Allelochemicals in the Leaves of the Aromatic Medicinal Tree Aegle marmelos (L.) Correa
Source: Plants (Basel). 2024 Feb 19;13(4):559. doi: 10.3390/plants13040559 (PMC10892298; doi:10.3390/plants13040559)
Supplement: Supplementary file 1 [file plants-13-00559-s001.zip › plants-2840250-supplementary.pdf]

Spectral Data of five compounds

Compound-**1** (umbelliferone); ^{1}H NMR (500 MHz, acetone-d_6) δ_H 7.86 (1H, d, *J* = 9.5), 7.51 (1H, d, *J* = 8.5), 6.84 (1H, dd, *J* = 8.5, 2.3), 6.75 (1H, d, *J* = 2.3), 6.16 (1H, d, *J* = 9.5); HRESIMS *m*/*z* 161.0242 [M−H]$^-$ (calcd for $C_9H_5O_3$ 161.0244).

Compound-**2** (*trans*-ferulic acid); ^{1}H NMR (500 MHz, CD_3OD + $CDCl_3$) δ_H 7.53 (1H, d, *J* = 15.8), 7.05 (1H, d, *J* = 1.9), 7.00 (1H, dd, *J* = 8.3, 1.9), 6.87 (1H, d, *J* = 8.3), 6.24 (1H, d, *J* = 15.8), δ_H 3.88 (3H, s); HRESIMS *m*/*z* 193.0506 [M−H]$^-$ (calcd for $C_{10}H_9O_4$ 193.0506).

Compound-**3** ((*E*)-4-hydroxycinnamic acid methyl ester); ^{1}H NMR (500 MHz, CD_3OD) δ_H 7.62 (1H, d, *J* = 16.0), 7.46 (2H, d, *J* = 8.6), 6.81 (2H, d, *J* = 8.6), 6.33 (1H, d, *J* = 16.0), 3.76 (3H, s); HRESIMS *m*/*z* 177.0555 [M−H]$^-$ (calcd for $C_{10}H_{10}O_3$ 177.0557).

Compound-**4** (*trans*-cinnamic acid); ^{1}H NMR (500 MHz, $CDCl_3$) δ_H 7.78 (1H, d, *J* = 16.0), 7.56 (2H, m), 7.43–7.39 (3H, m), 6.46 (1H, d, *J* = 16.0); HRESIMS *m*/*z* 147.0449 [M−H]$^-$ (calcd for $C_9H_8O_2$ 147.0452).

Compound-**5** (methyl (*E*)-3′-hydroxyl-4′-methoxycinnamate); ^{1}H NMR (500 MHz, $CDCl_3$) δ_H 7.60 (1H, d, *J* = 15.9), 7.13 (1H, d, *J* = 2.1), 7.03 (1H, dd, *J* = 8.3, 2.1), 6.84 (1H, d, *J* = 8.3), 6.29 (1H, d, *J* = 15.9), 3.93 (3H, s), 3.79 (3H, s); HRESIMS *m*/*z* 207.0660 [M−H]$^-$ (calcd for $C_{11}H_{11}O_4$ 207.0663).
